# Expression of Sclerostin in Osteoporotic Fracture Patients Is Associated with DNA Methylation in the CpG Island of the *SOST* Gene

**DOI:** 10.1155/2019/7076513

**Published:** 2019-01-08

**Authors:** Yanming Cao, Bin Wang, Ding Wang, Dongxiang Zhan, Caiyuan Mai, Peng Wang, Qiushi Wei, Yamei Liu, Haibin Wang, Wei He, Liangliang Xu

**Affiliations:** ^1^Department of Orthopedics, The Second Affiliated Hospital of Guangzhou Medical University, Guangzhou, China; ^2^Department of Orthopedics, People's Hospital of Sanshui, Foshan, China; ^3^Key Laboratory of Orthopaedics & Traumatology, The First Affiliated Hospital of Guangzhou University of Chinese Medicine, The First Clinical Medical College, Guangzhou University of Chinese Medicine, Guangzhou, China; ^4^Department of Obstetrics, Guangdong Women and Children's Hospital, Guangzhou 510010, China; ^5^Departments of Diagnostics of Traditional Chinese Medicine, Guangzhou University of Traditional Chinese Medicine, Guangzhou, Guangdong 510006, China; ^6^Laboratory of Orthopaedics & Traumatology, Lingnan Medical Research Center, Guangzhou University of Chinese Medicine, Guangzhou, China

## Abstract

**Purpose:**

*SOST* gene is one of the key factors in regulating bone absorption. Although there are reports showing diverse transcription factors, epigenetic modification could be responsible for regulating *SOST* gene expression. There is still little exploration on promoter methylation status of *SOST* gene in osteoporotic bone tissues. The aim of this study is to investigate the involvement of CpG methylation in regulation of SOST expression in patients with primary osteoporosis.

**Methods:**

The diagnosis of osteoporosis was established on the basis of dual energy X-ray absorptiometry to measure BMD. All femoral bone tissues were separated in surgeries. After extracting total RNA and protein, we checked the relative expression levels of SOST by quantitative real-time PCR and western blot. Also, immunohistochemical staining was performed to observe the expression of SOST protein in the bone samples. The genomic DNA of non-OPF (non-osteoporotic fracture bone tissues) and OPF (osteoporotic fracture bone tissues) were treated by bisulfite modification, and methylation status of CpG sites in the CpG island of *SOST* gene promoter was determined by DNA sequencing.

**Results:**

*SOST* gene expression in the non-OPF group was lower than that in OPF group. Bisulfite sequencing result showed that *SOST* gene promoter was slightly demethylated in the OPF group, as compared with non-OPF group.

**Conclusion:**

Our study demonstrated that DNA methylation influenced the transcriptional expression of *SOST* gene, which probably may play an important role in the pathogenesis of primary osteoporosis.

## 1. Introduction

Sclerostin (SOST) is the secreted glycoprotein encoded by the *SOST* gene. SOST mRNA and protein are specifically expressed in osteocytes which are the most prevalent cells in mineralized bone [1, 2]. It is a potent inhibitor of bone formation which antagonizes the canonical Wnt signaling by binding to Wnt coreceptors LRP-4, LRP-5, and LRP-6 [3, 4]. Mutations in the *SOST* gene are associated with disorders such as sclerosteosis and van Buchem disease characterized by increased bone mass [5, 6]. And the SOST knockout mice have a high bone mass phenotype characterized by significant increases in BMD (bone mineral density), bone volume, bone formation, and bone strength [7]. Since then, sclerostin has emerged as a key negative regulator of bone metabolism. A recent study suggests that sclerostin may have a catabolic action through promoting osteoclast formation and activity by osteocytes, in a RANKL-dependent manner [8]. Nowadays, anti-sclerostin antibodies are being tested to treat severe osteoporosis in clinical trials [9–11]. Also, the anti-sclerostin antibody has been successfully used to treat osteogenesis imperfecta in mouse models [11, 12]. Many factors have been identified to modulate SOST expression, such as BMPs (bone morphogenetic proteins), PTH (parathyroid hormone), TNF*α* (tumor necrosis factor-alpha), and mechanical forces [13–15].

DNA methylation can lead to variations in gene expression without changing its DNA sequence. It has been demonstrated that demethylation of the SOST promoter by 5-aza-2′-deoxycytidine (AzadC) induces a strong increase in SOST expression in MG63 osteosarcoma cell line, presumably by facilitating the binding of transcription factors to the proximal promoter [16]. Reppe et al. have also found there is correlation between sclerostin expression and DNA methylation in promoter of the *SOST* gene [17]. However, none of these studies have investigated the methylation status of the CpG island of *SOST* gene in bone tissues of patients with primary osteoporosis.

It has been reported that elevated serum sclerostin levels are associated with increased risk of hip fracture in older women [18]. However, on the other hand, conflicting result has been observed [17, 19]. So, it is very interesting and necessary to provide more evidences to demonstrate the expression of sclerostin in osteoporosis and its correlation with DNA methylation. Therefore, we explored the expression of sclerostin at both mRNA and protein levels in patients with osteoporotic fractures and normal fractured patients. In addition, bone biopsies were used for DNA methylation analysis to find out whether methylation status of the CpG island in *SOST* gene promoter was involved in regulating sclerostin expression.

## 2. Materials and Methods

### 2.1. Ethical Statement

16 primary osteoporosis patients with femoral neck/trochanter fractures (OPF, case group) and 16 patients with traumatic fractures (non-OPF, control group) were recruited in the Second Affiliated Hospital of Guangzhou Medical University. The bone mineral density (BMD) of the axial bone was measured by dual-energy X-ray absorptiometry (DEXA). Bone tissue samples were obtained during internal fixation surgery. The study was approved by the local ethics board and patients gave informed written consent. Patients with secondary osteoporosis, hip osteoarthritis, and pathological fracture due to nonosteoporosis were excluded.

### 2.2. Quantitative Real-Time RT-PCR (qRT-PCR)

Total RNA was extracted from fresh bone samples using Trizol (Invitrogen, USA) according to the manufacture's instruction. The qRT-PCR was performed as previously reported with minor revision [20]. The mRNA was reverse-transcribed to cDNA by the PrimeScript First Strand cDNA Synthesis Kit (TaKaRa). 5 *μ*l of total cDNA of each sample were amplified in a final volume of 25 *μ*l of reaction mixture containing Platinum SYBR Green, qPCR SuperMix-UDG ready-to-use reaction cocktail, and specific primers using the ABI StepOnePlus system (all from Applied Biosystems, CA, USA). The expression of target gene was normalized to that of GAPDH gene which was shown to be stable in this study. Relative gene expression was calculated with the 2^-△CT^ formula. The sequences of the primers were shown in Supplementary Table 1.

### 2.3. DNA Isolation and Bisulfite Treatment

Genomic DNA was isolated from fresh bone samples. Briefly, the samples were digested with proteinase K, extracted with phenol/chloroform/isoamyl alcohol (25 : 24 : 1), precipitated with ethanol, and resuspended in TE buffer (0.1 M Tris, 1 mM Na_2_EDTA, pH 7.5). Bisulfite modification was done as described previously [21, 22]. Briefly, about 2 *μ*g of genomic DNA was denatured by NaOH (final concentration, 0.2 mol/l) for 10 min at 37°C. Hydroquinone and sodium hydroxide were added, and samples were incubated at 50°C for 16 h. Modified DNA was purified using Wizard DNA Clean-Up System following the manufacturer's instructions (Promega) and eluted into 50 *μ*l water. DNA was treated with NaOH (final concentration, 0.3 mol/l) for 5 min at room temperature, ethanol precipitated, and resuspended in 20 *μ*l water. Modified DNA was used immediately or stored at −20°C.

### 2.4. Bisulfite Sequencing

Bisulfite-modified genomic DNA was amplified by PCR. All PCRs were done using KAPA2G™ Fast HotStart DNA Polymerase. The sequences of primers used for the bisulfite sequencing analysis were shown in Supplementary Table 2. PCR products were run on 1.5% agarose gels and bands were excised using TaKaRa MiniBEST Agarose Gel DNA Extraction Kit following the manufacturer's instructions (TaKaRa). Purified bands were cloned using pMD™19-T Vector Cloning Kit following the manufacturer's instructions (TaKaRa). Colonies were selected and grown overnight in Luria-Bertani medium containing ampicillin (100 *μ*g/ml) with shaking at 37°C. Plasmid DNA was isolated using TaKaRa MiniBEST Agarose Gel DNA Extraction Kit following the manufacturer's instructions (TaKaRa). Plasmids were sequenced using the M13 universal reverse primer (BGI).

### 2.5. Histology and Immunohistochemistry

Immunohistochemical staining was performed as previously described [23, 24]. The samples were washed in PBS, fixed in 4% paraformaldehyde, decalcified, dehydrated, and embedded in paraffin. Sections were cut at a thickness of 5 *μ*m and were stained with H&E after deparaffination. Endogenous peroxidase activity was quenched with 3% hydrogen peroxide for 20 minutes at room temperature. Antigen retrieval was then performed with citrate buffer at 80°C for 10 minutes for immunohistochemistry detection. Primary antibody against SOST protein (1 : 100; sc-365797, Santa Cruz, CA, USA) was used. Donkey anti-goat IgG horseradish peroxidase- (HRP-) conjugated secondary antibody was then added for an hour, followed by 3,3′ diaminobenzidine tetrahydrochloride (Dako, Glostrup, Denmark) in the presence of H_2_O_2_ for signal detection of SOST. Afterward, the sections were rinsed, counterstained in hematoxylin, dehydrated with graded ethanol and xylene, and mounted with p-xylene-bis-pyridinium bromide (DPX) permount (Sigma-Aldrich, St. Louis, MO, USA). Primary antibody was replaced with blocking solution in the negative controls. All incubation times and conditions were strictly controlled. The sections were examined under light microscopy (DMRXA2, Leica Microsystems Wetzlar GmbH, Germany).

### 2.6. Data Analysis

All experiments were performed at least 3 times. All data were expressed as the mean ± SD. The data were analyzed by nonparametric test (Mann-Whitney) using SPSS (version 16.0; Chicago, IL, USA). *p* < 0.05 was regarded as statistically significant.

## 3. Results

### 3.1. Expression of *SOST* Gene in Patients with Osteoporotic Fracture

**Figure 1:**
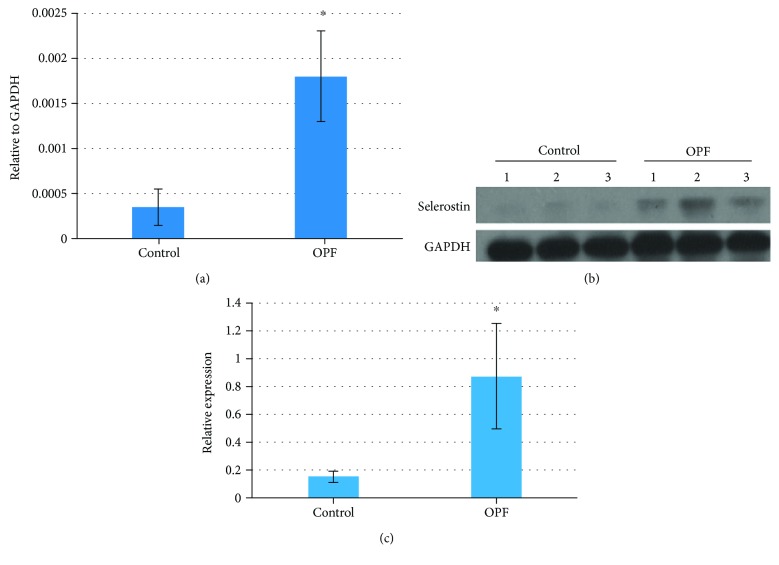
Expression level of SOST in bone tissue samples. (a) Total RNA was extracted from bone tissues of patients with OPF or non-OPF. GAPDH was used as an internal control. The data are expressed as mean ± SD (*n* = 16). ^∗^ *p* < 0.05. (b) Total proteins extracted from bone tissues of patients with OPF or non-OPF were analyzed by western blot using anti-SOST antibody. *β*-Actin was used as loading control (*n* = 3). (c) The protein levels of SOST in control and OPF groups were quantified using ImageJ software. Data is presented as mean ± SD (*n* = 3, *p* < 0.05).

16 osteoporotic patients with femoral neck/trochanter fractures (OPF, case group) and 16 normal patients with traumatic fractures (non-OPF, control group) were recruited in the Second Affiliated Hospital of Guangzhou Medical University. The control group had normal BMD but were somewhat younger which is inevitable. The mRNA level of SOST was compared by quantitative real-time PCR. The result showed that SOST mRNA expression level was significantly increased in the OPF group (Figure 1(a), *n* = 16). We then isolated total proteins from bone tissues of 3 patients in each group and checked the protein level of sclerostin by western blot. We found that the expression level of sclerostin was much higher in the OPF group (Figures 1(b) and 1(c)), which is consistent with the quantitative real-time PCR result.

### 3.2. Detection of SOST in the Human Bone by Immunohistochemical Staining

**Figure 2:**
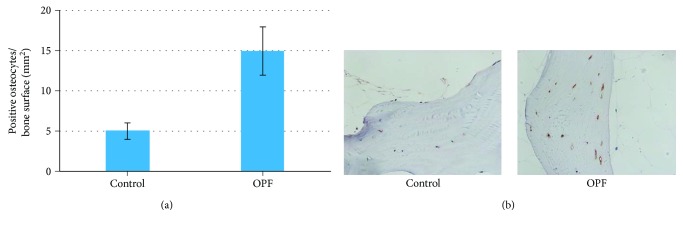
Detection of SOST in bone samples by immunohistochemical staining. Bone samples of OPF and non-OPF were decalcified and sectioned. Antisclerostin antibody was used for immunohistochemical staining. SOST was specifically expressed in osteocytes. (a) The number of SOST-positive osteocytes was counted. Data is presented as mean ± SD (*n* = 3, *p* < 0.05). (b) Typical images of immunohistochemical staining of SOST in control and OPF groups.

In order to observe the expression of SOST protein in the bone samples, we further conducted immunohistochemical staining. As expected, the staining of bone samples with a specific sclerostin antibody confirmed that SOST was highly and specifically expressed in osteocytes in OPF group (Figures 2(a) and 2(b)).

### 3.3. Methylation of CpG Island in SOST Promoter in Human Bone Tissues

**Figure 3:**
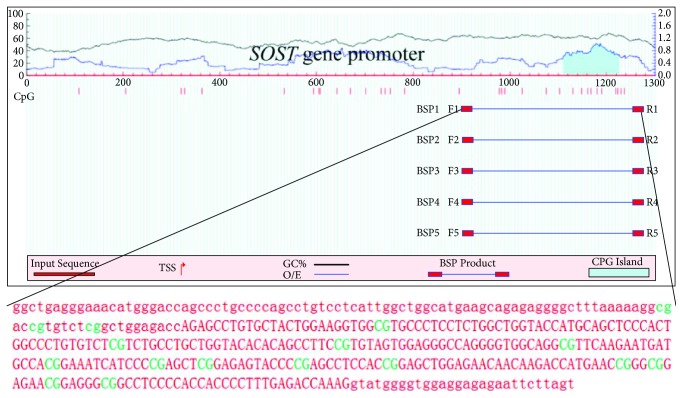
Schematic figure indicates 16 CpG sites in CpG island of the *SOST* gene promoter. Exons in upper case, everything else in lower case. The CpG sites were shown in green.

**Figure 4:**
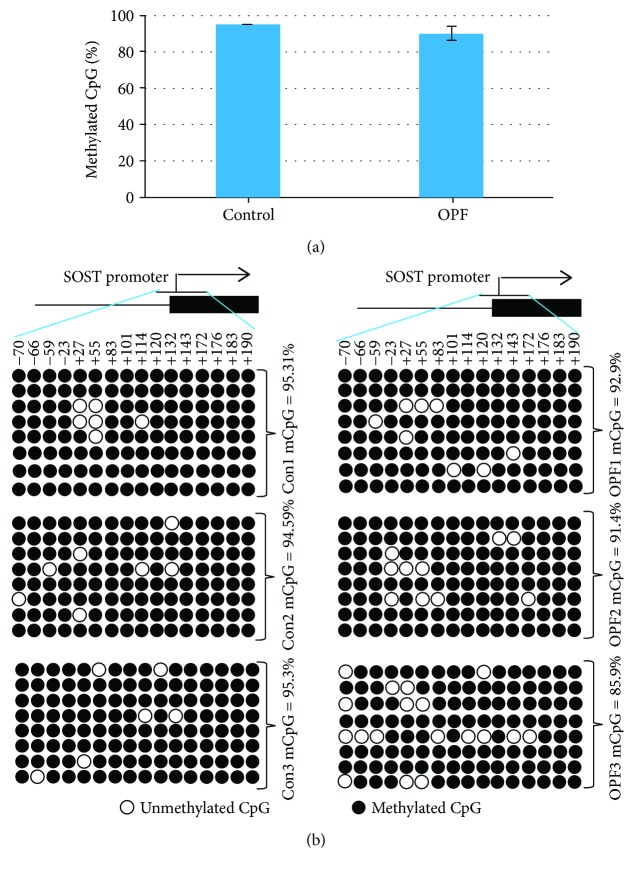
Epigenetic regulation of SOST in bone tissues. DNA methylation status of SOST promoter in three non-OPF and three OPF samples using sodium bisulfite sequencing. Each PCR product was subcloned and subjected to nucleotide sequencing analysis. (a) The percentage of methylated CpG sites in SOST promoter was calculated based on the BSP sequencing result. (b) BSP sequencing result of methylated CpG sites in each samples. Sequenced clones were depicted by filled (methylated) and open (unmethylated) circles for each CpG site.

MethPrimer (http://www.urogene.org/cgi-bin/methprimer2/MethPrimer.cgi) was used to analyze a length of the CpG-rich region around the transcription start site of *SOST* gene promoter. One CpG island containing 16 CpG sites was revealed in the *SOST* gene promoter (Figure 3). After bisulfite treatment of DNA obtained from bone tissues of OPF and non-OPF patients, we calculated the percentage of methylated CpG site in the total 16 CpG sites in SOST promoter. We found that *SOST* gene promoter was hypermethylated in both OPF and non-OPF groups. But the methylation ratio was slightly lower in the OPF group, which means demethylation of CpG sites in *SOST* gene promoter might contribute to its increased expression (Figures 4(a) and 4(b)).

To sum up, our data demonstrated that epigenetic regulation, or rather, DNA methylation in the bone metabolism disorder patients regulated *SOST* gene expression, which contributes to the occurrence of osteoporosis.

## 4. Discussion

The investigation about the relationship between methylation level of CpG-rich region and gene expression has been emerging constantly. There is increasing experimental evidence on the potential role of DNA methylation in neoplastic disorders [25] and in metabolic bone disease [26]. Nevertheless, little is known about the specific relationship between DNA methylation and *SOST* gene expression in patients with primary osteoporosis.

In the present study, we demonstrated that the expression level of *SOST* gene was increased in bone tissues obtained from patients with OPF. We found that 16 CpG sites in the CpG island of *SOST* gene promoter were hypermethylated in both groups, but the level of methylation in the OPF group was slightly decreased. These results demonstrated that DNA demethylation could increase SOST expression, which was consistent with the quantitative real-time PCR data. This finding strongly suggested the *SOST* gene promoter demethylation may be an important inducer for pathogenesis of osteoporosis.

DNA methylation has been proved to be involved in numerous biological events (e.g., embryonic development, parental imprinting genes, transposon silencing, X inactivation, and cancer), and it concerns about 70–80% of CpGs in mammalian DNA [27–29]. Generally, low levels or a lack of DNA methylation in the promoter region is correlated with activation of gene expression, as the configuration of chromatin favors the interaction of DNA with transcription complexes. By contrast, methylation of CpG islands in gene promoters is correlated with gene silencing [30]. Up to now, evolving evidence has suggested that DNA methylation may be involved in age-related diseases and bone biology [31]. Our previous studies have found that DNA methylation plays an essential role in determining the fate of mesenchymal stem cells [24, 32]. In this study, we explored whether *SOST* gene expression in OPF patients was influenced by the epigenetic modulation. As mentioned in the introduction, DNA methylation is linked with transcriptional silencing of associated genes [33]. It was reported that researchers had used an integrated genomic reporter system to insert DNA methylation specifically distal to the start site of transcription and found that the reduced expression of the reporter was not caused by the effects of DNA methylation on initiation of transcription or promoter clearance but with RNA polymerase II and chromatin accessibility reduction in comparison to the unmethylated control plasmid [34].

Three classes of DNA methyl transferases (DNMTs) are involved in DNA methylation, including DNMT1, DNMT2, and the DNMT3A/3B/3L [35, 36]. For example, DNMT1, composed of a large regulator N-terminal region (1000 aa) and a small catalytic C-terminal region, mainly catalyzes DNA methylation inheritance activity [37, 38]; DNMT3A and DNMT3B are the enzymes predominantly associated with de novo DNA methylation [39]. Interestingly, apart from the CpG island investigated in the present study, other *cis*-acting elements have also been identified to regulate SOST expression. For example, the enhancer at the 35 kb downstream of SOST has been found to function in *cis* to enhance SOST transcription [40]. In addition, an evolutionarily conserved region (ECR5) has also been identified to drive SOST expression in vitro and in vivo [41]. Recent advances in genome-wide methylation methods have provided the means to identify differentially methylated genes, methylation signatures which have the potential to be used as biomarkers. SOST is an important player in the pathogenesis of osteoporosis [42, 43]; the finding that its expression is associated with DNA methylation could make it a useful biomarker of diagnosis of osteoporosis.

In a word, we found that the percentage of methylated CpG sites in the CpG island of SOST gene was slightly decreased in the patients with OPF, implying that methylation status in CpG island of *SOST* gene have influenced its expression level in patients with OPF. And the pathogenesis of osteoporosis may be partially attributed to the demethylation of *SOST* gene.

## Data Availability

The data used to support the findings of this study are available from the corresponding author upon request.

## Supplementary Materials

Supplementary 1 Table 1: primer sequences for qRT-PCR.

Supplementary 2 Table 2: the sequences of SOST primer used for bisulfite sequencing PCR.

## References

[1] Winkler D. G., Sutherland M. K., Geoghegan J. C. (2003). Osteocyte control of bone formation via sclerostin, a novel BMP antagonist. *The EMBO Journal*.

[2] Poole K. E. S., van Bezooijen R. L., Loveridge N. (2005). Sclerostin is a delayed secreted product of osteocytes that inhibits bone formation. *The FASEB Journal*.

[3] ten Dijke P., Krause C., de Gorter D. J. J., Löwik C. W. G. M., van Bezooijen R. L. (2008). Osteocyte-derived sclerostin inhibits bone formation: Its role in bone morphogenetic protein and Wnt signaling. *The Journal of Bone and Joint Surgery-American Volume*.

[4] Li X., Zhang Y., Kang H. (2005). Sclerostin binds to LRP5/6 and antagonizes canonical Wnt signaling. *The Journal of Biological Chemistry*.

[5] Brunkow M. E., Gardner J. C., van Ness J. (2001). Bone dysplasia sclerosteosis results from loss of the SOST gene product, a novel cystine knot-containing protein. *American Journal of Human Genetics*.

[6] Staehling-Hampton K., Proll S., Paeper B. W. (2002). A 52-kb deletion in the SOST-MEOX1 intergenic region on 17q12-q21 is associated with van Buchem disease in the Dutch population. *American Journal of Medical Genetics*.

[7] Li X., Ominsky M. S., Niu Q. T. (2008). Targeted deletion of the sclerostin gene in mice results in increased bone formation and bone strength. *Journal of Bone and Mineral Research*.

[8] Wijenayaka A. R., Kogawa M., Lim H. P., Bonewald L. F., Findlay D. M., Atkins G. J. (2011). Sclerostin stimulates osteocyte support of osteoclast activity by a RANKL-dependent pathway. *PLoS One*.

[9] McClung M. R., Grauer A., Boonen S. (2014). Romosozumab in postmenopausal women with low bone mineral density. *New England Journal of Medicine*.

[10] McClung M. R., Chines A., Brown J. P. (2014). Effects of 2 years of treatment with romosozumab followed by 1 year of denosumab or placebo in postmenopausal women with low bone mineral density. *Journal of Bone and Mineral Research*.

[11] McClung M. R., Grauer A., Boonen S. (2013). OP0248 Inhibition of sclerostin with romosozumab in postmenopausal women with low bone mineral density: phase 2 trial results. *Annals of the Rheumatic Diseases*.

[12] Grafe I., Alexander S., Yang T. (2016). Sclerostin antibody treatment improves the bone phenotype of Crtap−/− mice, a model of recessive osteogenesis imperfecta. *Journal of Bone and Mineral Research*.

[13] Leupin O., Kramer I., Collette N. M. (2007). Control of the SOST bone enhancer by PTH using MEF2 transcription factors. *Journal of Bone and Mineral Research*.

[14] Papanicolaou S. E., Phipps R. J., Fyhrie D. P., Genetos D. C. (2009). Modulation of sclerostin expression by mechanical loading and bone morphogenetic proteins in osteogenic cells. *Biorheology*.

[15] Vincent C., Findlay D. M., Welldon K. J. (2009). Pro-inflammatory cytokines tnf-related weak inducer of apoptosis (TWEAK) and TNFα induce the mitogen-activated protein kinase (MAPK)-dependent expression of sclerostin in human osteoblasts. *Journal of Bone and Mineral Research*.

[16] Delgado-Calle J., Sañudo C., Bolado A. (2012). DNA methylation contributes to the regulation of sclerostin expression in human osteocytes. *Journal of Bone and Mineral Research*.

[17] Reppe S., Noer A., Grimholt R. M. (2015). Methylation of bone SOST, its mRNA, and serum sclerostin levels correlate strongly with fracture risk in postmenopausal women. *Journal of Bone and Mineral Research*.

[18] Arasu A., Cawthon P. M., Lui L. Y. (2012). Serum sclerostin and risk of hip fracture in older Caucasian women. *The Journal of Clinical Endocrinology & Metabolism*.

[19] Dovjak P., Dorfer S., Föger-Samwald U., Kudlacek S., Marculescu R., Pietschmann P. (2014). Serum levels of sclerostin and Dickkopf-1: effects of age, gender and fracture status. *Gerontology*.

[20] Xu L., Huang S., Hou Y. (2015). Sox11-modified mesenchymal stem cells (MSCs) accelerate bone fracture healing: Sox11 regulates differentiation and migration of MSCs. *The FASEB Journal*.

[21] Zinn R. L., Pruitt K., Eguchi S., Baylin S. B., Herman J. G. (2007). hTERT is expressed in cancer cell lines despite promoter DNA methylation by preservation of unmethylated DNA and active chromatin around the transcription start site. *Cancer Research*.

[22] Yannarelli G., Pacienza N., Cuniberti L., Medin J., Davies J., Keating A. (2013). Brief report: the potential role of epigenetics on multipotent cell differentiation capacity of mesenchymal stromal cells. *Stem Cells*.

[23] Rui Y. F., Lui P. P. Y., Lee Y. W., Chan K. M. (2012). Higher BMP receptor expression and BMP-2-induced osteogenic differentiation in tendon-derived stem cells compared with bone-marrow-derived mesenchymal stem cells. *International Orthopaedics*.

[24] Xu L., Liu Y., Sun Y. (2017). Tissue source determines the differentiation potentials of mesenchymal stem cells: a comparative study of human mesenchymal stem cells from bone marrow and adipose tissue. *Stem Cell Research & Therapy*.

[25] Esteller M. (2008). Epigenetics in cancer. *The New England Journal of Medicine*.

[26] Reppe S., Lien T. G., Hsu Y. H. (2017). Distinct DNA methylation profiles in bone and blood of osteoporotic and healthy postmenopausal women. *Epigenetics*.

[27] Guibert S., Weber M. (2013). Functions of DNA methylation and hydroxymethylation in mammalian development. *Current Topics in Developmental Biology*.

[28] Smallwood S. A., Kelsey G. (2012). De novo DNA methylation: a germ cell perspective. *Trends in Genetics*.

[29] Cotton A. M., Price E. M., Jones M. J., Balaton B. P., Kobor M. S., Brown C. J. (2015). Landscape of DNA methylation on the X chromosome reflects CpG density, functional chromatin state and X-chromosome inactivation. *Human Molecular Genetics*.

[30] Yoo C. B., Jones P. A. (2006). Epigenetic therapy of cancer: past, present and future. *Nature Reviews. Drug Discovery*.

[31] Boyd-Kirkup J. D., Green C. D., Wu G., Wang D., Han J. D. J. (2013). Epigenomics and the regulation of aging. *Epigenomics*.

[32] Rui Y., Xu L., Chen R. (2015). Epigenetic memory gained by priming with osteogenic induction medium improves osteogenesis and other properties of mesenchymal stem cells. *Scientific Reports*.

[33] Hsieh C. L. (1997). Stability of patch methylation and its impact in regions of transcriptional initiation and elongation. *Molecular and Cellular Biology*.

[34] Lorincz M. C., Dickerson D. R., Schmitt M., Groudine M. (2004). Intragenic DNA methylation alters chromatin structure and elongation efficiency in mammalian cells. *Nature Structural & Molecular Biology*.

[35] Ludwig A. K., Zhang P., Cardoso M. C. (2016). Modifiers and readers of DNA modifications and their impact on genome structure, expression, and stability in disease. *Frontiers in Genetics*.

[36] Hashimoto H., Vertino P. M., Cheng X. (2010). Molecular coupling of DNA methylation and histone methylation. *Epigenomics*.

[37] Hervouet E., Vallette F. M., Cartron P. F. (2010). Dnmt1/transcription factor interactions: an alternative mechanism of DNA methylation inheritance. *Genes & Cancer*.

[38] Kar S., Deb M., Sengupta D. (2014). An insight into the various regulatory mechanisms modulating human DNA methyltransferase 1 stability and function. *Epigenetics*.

[39] Jin B., Robertson K. D. (2013). DNA methyltransferases, DNA damage repair, and cancer. *Epigenetic Alterations in Oncogenesis*.

[40] Balemans W., Patel N., Ebeling M. (2002). Identification of a 52 kb deletion downstream of the SOST gene in patients with van Buchem disease. *Journal of Medical Genetics*.

[41] Loots G. G., Kneissel M., Keller H. (2005). Genomic deletion of a long-range bone enhancer misregulates sclerostin in van Buchem disease. *Genome Research*.

[42] Chang M. K., Kramer I., Keller H. (2014). Reversing LRP5-dependent osteoporosis and SOST deficiency-induced sclerosing bone disorders by altering WNT signaling activity. *Journal of Bone and Mineral Research*.

[43] Zhou P. R., Xu X. J., Zhang Z. L. (2015). SOST polymorphisms and response to alendronate treatment in postmenopausal Chinese women with osteoporosis. *Pharmacogenomics*.

