# Supplementary material for: Expression of Sclerostin in Osteoporotic Fracture Patients Is Associated with DNA Methylation in the CpG Island of the SOST Gene
Source: Int J Genomics. 2019 Jan 8;2019:7076513. doi: 10.1155/2019/7076513 (PMC6341240; doi:10.1155/2019/7076513)
Supplement: Supplementary 1 — Table 1: primer sequences for qRT-PCR. [file 7076513.f1.docx]

**Supplementary Table 1**. **Primer sequences for qRT-PCR.**

| **Primer** | **Sequence (5’-3’)** |
| --- | --- |
| GADPH-Fwd  GADPH-Rev  SOST-Fwd  SOST-Rev | GGCATGGACTGTGGTCATGAG  TGCACCACCAACTGCTTAGC  GGCAGCTGTACTCGGACAC  TGATGCCACGGAAATCATC |

The GAPDH and SOST primers were used for quantitative real time RT-PCR to detect the relative expression level of SOST in bone tissue samples obtained from fractured patients.
