# Supplementary material for: Expression of Sclerostin in Osteoporotic Fracture Patients Is Associated with DNA Methylation in the CpG Island of the SOST Gene
Source: Int J Genomics. 2019 Jan 8;2019:7076513. doi: 10.1155/2019/7076513 (PMC6341240; doi:10.1155/2019/7076513)
Supplement: Supplementary 2 — Table 2: the sequences of SOST primer used for bisulfite sequencing PCR. [file 7076513.f2.docx]

**Supplementary Table 2**. **The sequences of SOST primer used for bisulfite sequencing PCR.**

| **Primer Sequence (5’ - 3’)** | |
| --- | --- |
| SOST-Me-Fwd  SOST-Me-Rev | GGTTGAGGGAAATATGGGATTAG  CCCATACCTTTAATCTCAAAAA |

The SOST primer was used for bisulfite sequencing PCR to check the methylation status of CpG sites in SOST gene promoter in bone tissue samples obtained from fractured patients.
